# Computed tomography detected pyelovenous backflow associated with complete ureteral obstruction

**DOI:** 10.1002/iju5.12117

**Published:** 2019-09-01

**Authors:** Takeshi Sano, Noriatsu Ichiba, Kimihiko Masui, Takao Haitani, Keita Takimoto, Yoichiro Kajita, Yasumasa Shichiri

**Affiliations:** ^1^ Department of Urology Otsu City Hospital Otsu Japan; ^2^ Department of Urology Kyoto University Graduate School of Medicine Kyoto Japan; ^3^ Department of Radiology Otsu City Hospital Otsu Japan; ^4^ Kajita Urological Clinic Kyoto Japan

**Keywords:** contrast‐enhanced computed tomography, hydronephrosis, pyelovenous backflow, ureteral obstruction, ureteroneocystostomy

## Abstract

**Introduction:**

Pyelovenous backflow is a rare condition resulting from an increase in pressure in the renal pelvis due to urinary obstruction.

**Case presentation:**

A 49‐year‐old woman developed high‐grade fever and right‐sided hydronephrosis after undergoing hysterectomy. Although the hydronephrosis was mild, retrograde pyelography revealed complete obstruction of the right ureter. Excretory phase scans of contrast‐enhanced computed tomography showed pyelovenous backflow, which presumably decompressed the hydronephrosis. The pyelovenous backflow immediately disappeared after ureteroneocystostomy.

**Conclusion:**

We were presented with a patient showing pyelovenous backflow detected by contrast‐enhanced computed tomography, which completely disappeared after ureteral obstruction release.

**Abbreviations & Acronyms**
CT: computed tomography
IVC: inferior vena cava
PVB: pyelovenous backflow
RP: retrograde pyelography

**Keynote message**

We present a rare case of PVB, the abnormal urinary flow from the renal pelvis to the renal vein, detected by contrast‐enhanced CT. PVB presumably reduced the extent of hydronephrosis and delayed the diagnosis of complete ureteral obstruction. This case may demonstrate the importance of contrast‐enhanced CT in revealing the cause of persistent hydronephrosis, even in a mild case.

## Introduction

Hydronephrosis is commonly caused by ureteral obstruction, with the grade of condition varying based on the severity of the obstruction, and is usually diagnosed by sonography or CT without difficulty. PVB, the abnormal urinary flow from the renal pelvis to the renal vein, occasionally occurs when the renal pelvis is under high pressure.^1^ This backflow lowers the intrapelvic pressure, reducing the extent of the hydronephrosis, and makes the diagnosis of ureteral obstruction more difficult.^2^ The backflow has been detected with RP in most cases.^3^ Here, we present a rare case of a patient wherein PVB delayed the diagnosis of complete ureteral obstruction. PVB, however, was eventually detected using excretory phase images of contrast‐enhanced CT.

## Case presentation

**Figure 1:**
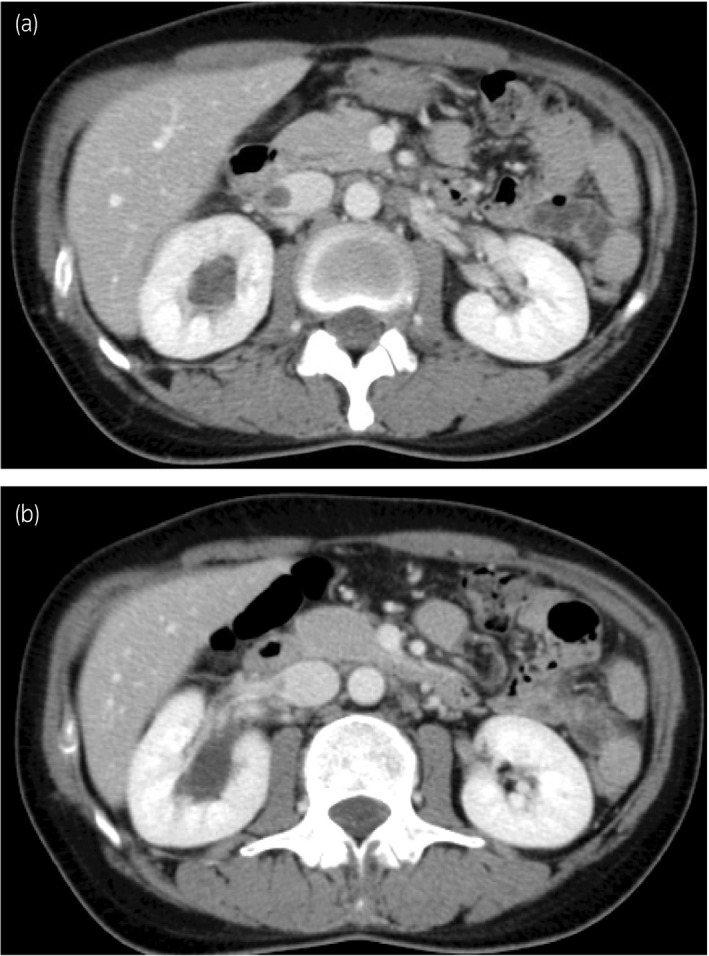
Axial images during the nephrographic phase of contrast‐enhanced CT before ureteroneocystostomy. (a) Mild, right hydronephrosis with homogeneous renal parenchymal enhancement and the low‐density lesion in the IVC, wherein densities in both lesions are very similar. (b) The low‐density lesion in the IVC and dilated renal pelvis appears to be continuous through the renal vein.

**Figure 2:**
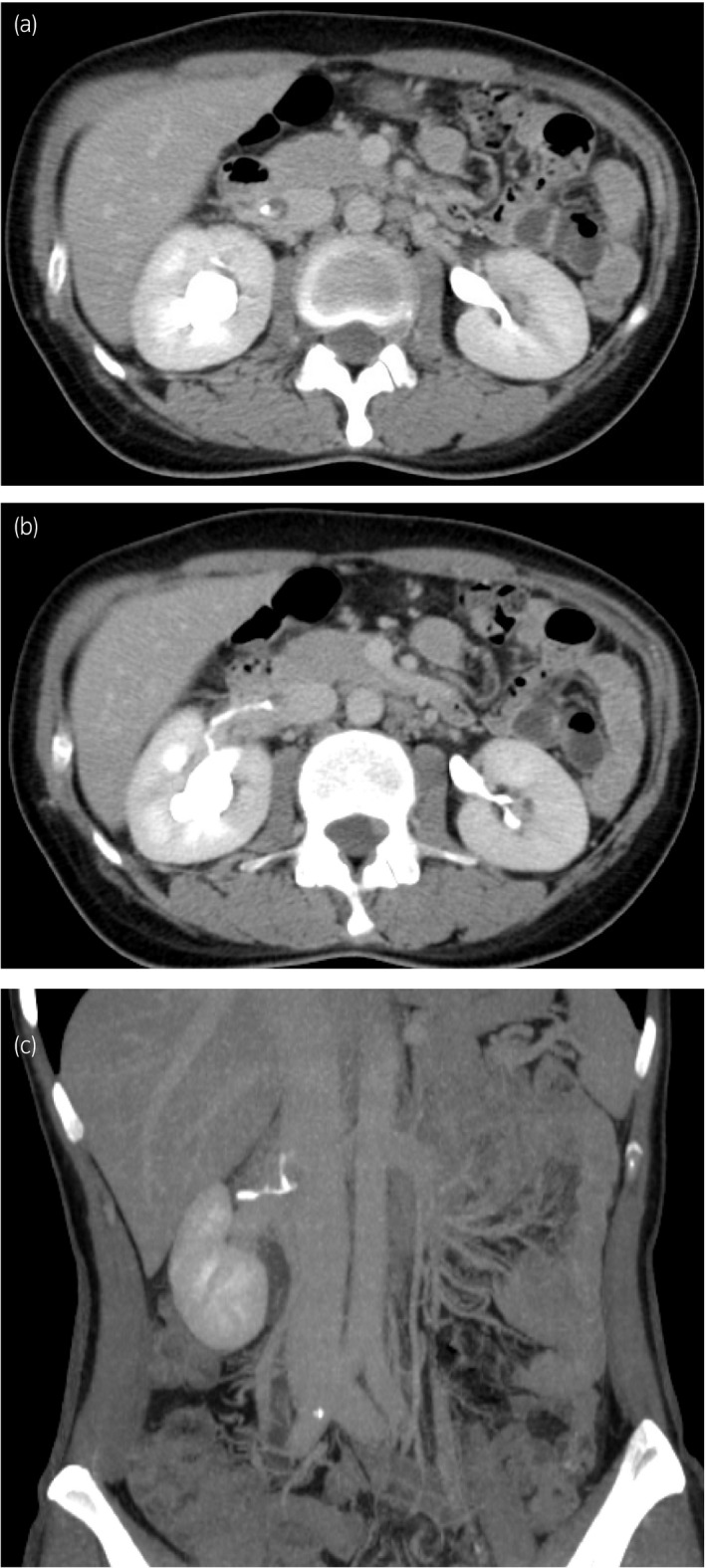
Axial images (a,b) and a coronal reconstructive image (c) during the excretory phase of contrast‐enhanced CT before ureteroneocystostomy. (a) The contrast medium flowed into the low‐density lesion in the IVC, and the renal pelvis was filled with contrast medium. (b) A thin, meandering backflow of the contrast medium from the right renal pelvis through the renal vein. (c) Contrast medium going cephalad along the IVC.

A 49‐year‐old woman, with uterine myoma, underwent a laparoscopically assisted vaginal hysterectomy in August 2011. The surgery was converted to open surgery because of the severe adhesion between the myoma and the right ureter. On postoperative day 2, she complained of right flank pain, and a sonogram revealed mild right‐sided hydronephrosis. The patient was referred to the urology department, and it was speculated that the right ureter was edematous due to damage that occurred during the hysterectomy, which led to the mild hydronephrosis. Thus specific interventions were not instituted at that moment. The patient developed fever of >38°C on postoperative day 3, and RP revealed complete obstruction of the right ureter at the ureterovesical junction where neither a guidewire nor the contrast medium passed through the obstruction at all. We performed contrast‐enhanced CT to identify the cause of the fever and ureteral obstruction. The ureteral obstruction at the ureterovesical junction was approximately 3.5 cm long. Nephrographic phase images showed that the right kidney was enhanced to the same degree as the left kidney despite the complete obstruction of the right ureter (Fig. 1). Excretory phase images depicted backflow from the renal pelvis via the renal vein (Fig. 2). PVB was determined to be responsible for the decompression of the hydronephrosis, such that the contrast enhancement of the renal parenchyma was preserved despite the complete ureteral obstruction. Both venous and excretory phase images also revealed a low‐density lesion in the IVC (Figs 1a,2a,c). We suspected the lesion to be a thrombus and placed a temporal IVC filter from the internal jugular vein to prevent it from traveling to the lungs. Ureteroneocystostomy was performed 13 days after hysterectomy, and a CT scan performed 4 days after the procedure revealed that the hydronephrosis, PVB, and the low‐density lesion in the IVC had completely disappeared without any thrombolytic therapy. The serum creatinine level gradually increased up to 1.17 mg/dL after the hysterectomy and went back to the baseline level of 0.52 mg/dL 5 days after the ureteroneocystostomy.

## Discussion

The resorption of urine, caused by increased intrapelvic pressure, is termed “pyelorenal backflow,” and occurs via the following five pathways: PVB, pyelolymphatic, pyelotubular, pyelointerstitial, and pyelosinus.^4^ They are considered to be compensatory mechanisms that allow the kidney to continue urine excretion.^5^ Among them, PVB is hypothesized to result from the veno‐caliceal fistula or the synchronous rupture of a vein within the renal calyceal fornix.^6^, ^7^

Contrast‐enhanced CT is considered an alternative tool for identifying PVB in cases where RP is not applicable. Nemeth *et al*. reported the first case where excretory phase CT revealed PVB flowing into the IVC in a patient with an abdominopelvic abscess after a cesarean section delivery.^3^ Durhan *et al*. recently presented a case of pyelolymphatic backflow detected by a CT scan, in which the backflow showed thread‐like structures enhanced by contrast medium surrounding the renal pelvis and IVC.^2^ The findings of the CT in the present case appear to be PVB, not pyelolymphatic backflow. Nemeth *et al*. indicated that the patient had simultaneously developed a thrombus in the IVC and speculated that the thrombus slowed the backward flow, making the capture of the reflux on CT possible.^3^ Initially, we also suspected the low‐density lesion in the IVC to be a thrombus. Retrospectively, however, we speculate that the low‐density lesion was not a thrombus, but rather PVB before the contrast medium reached the urinary tract, for a few reasons. First, the low‐density lesion in the IVC and the dilated renal pelvis appeared to be connected with each other (Fig. 1b), and their density was very similar (Fig. 1a). The ranges of Hounsfield unit values of the CT in the low‐density area and the renal pelvis were between 47 and 54, and 45 and 53, respectively. Second, the contrast medium flowed into the low‐density lesion in the IVC (Fig. 2a), which almost rules out the possibility of a thrombus. Finally, the low‐density lesion disappeared immediately without any thrombolytic therapy after ureteroneocystostomy.

Although almost all backflows are temporally observed during RP and do not affect patients’ clinical courses, several cases that have influenced the clinical course have been reported. Chen *et al*. reported that an anastomotic stricture, related to ureteroneocystostomy during a renal transplant, produced PVB and renal failure.^5^ In their case, the patient's serum creatinine level rapidly increased, and an ultrasonography of the kidney graft detected a distended renal pelvis and ureter, whereas diuretic renogram showed good kidney perfusion, and renal biopsy did not reveal any evidence of rejection. Percutaneous nephrostomy was finally performed, and the PVB was diagnosed with nephrostogram. The authors alert clinicians to the possibility that PVB might create a pitfall by producing false‐negative results when interpreting the results of diuretic renogram.^5^ Our report shows another case wherein PVB decreased the grade of hydronephrosis, leading to a delay of the diagnosis of complete ureteral obstruction, and suggests that contrast‐enhanced CT can be a powerful tool to identify PVB in a patient with a persistent hydronephrosis.

## Conflict of interest

The authors declare no conflict of interest.
